# RcaE-Dependent Regulation of Carboxysome Structural Proteins Has a Central Role in Environmental Determination of Carboxysome Morphology and Abundance in *Fremyella diplosiphon*

**DOI:** 10.1128/mSphere.00617-17

**Published:** 2018-01-24

**Authors:** Brandon A. Rohnke, Shailendra P. Singh, Bagmi Pattanaik, Beronda L. Montgomery

**Affiliations:** a Department of Energy—Plant Research Laboratory, Michigan State University, Plant Biology Laboratories, East Lansing, Michigan, USA; b Department of Biochemistry and Molecular Biology, Michigan State University, East Lansing, Michigan, USA; c Department of Microbiology & Molecular Genetics, Michigan State University, East Lansing, Michigan, USA; University of Iowa

**Keywords:** carbon-concentrating mechanism (CCM), carboxysome, cyanobacteria, light signaling, photomorphogenesis, photosynthesis

## Abstract

Carboxysomes are central to the carbon dioxide-concentrating mechanism (CCM) and carbon fixation in cyanobacteria. Although the structure is well understood, roles of environmental cues in the synthesis, positioning, and functional tuning of carboxysomes have not been systematically studied. *Fremyella diplosiphon* is a model cyanobacterium for assessing impacts of environmental light cues on photosynthetic pigmentation and tuning of photosynthetic efficiency during complementary chromatic acclimation (CCA), which is controlled by the photoreceptor RcaE. Given the central role of carboxysomes in photosynthesis, we investigated roles of light-dependent RcaE signaling in carboxysome structure and function. A Δ*rcaE* mutant exhibits altered carboxysome size and number, *ccm* gene expression, and carboxysome protein accumulation relative to the wild-type (WT) strain. Several Ccm proteins, including carboxysome shell proteins and core-nucleating factors, overaccumulate in Δ*rcaE* cells relative to WT cells. Additionally, levels of carboxysome cargo RuBisCO in the Δ*rcaE* mutant are lower than or unchanged from those in the WT strain. This shift in the ratios of carboxysome shell and nucleating components to the carboxysome cargo appears to drive carboxysome morphology and abundance dynamics. Carboxysomes are also occasionally mislocalized spatially to the periphery of spherical mutants within thylakoid membranes, suggesting that carboxysome positioning is impacted by cell shape. The RcaE photoreceptor links perception of external light cues to regulating carboxysome structure and function and, thus, to the cellular capacity for carbon fixation.

**IMPORTANCE** Carboxysomes are proteinaceous subcellular compartments, or bacterial organelles, found in cyanobacteria that consist of a protein shell surrounding a core primarily composed of the enzyme ribulose-1,5-biphosphate carboxylase/oxygenase (RuBisCO) that is central to the carbon dioxide-concentrating mechanism (CCM) and carbon fixation. Whereas significant insights have been gained regarding the structure and synthesis of carboxysomes, limited attention has been given to how their size, abundance, and protein composition are regulated to ensure optimal carbon fixation in dynamic environments. Given the centrality of carboxysomes in photosynthesis, we provide an analysis of the role of a photoreceptor, RcaE, which functions in matching photosynthetic pigmentation to the external environment during complementary chromatic acclimation and thereby optimizing photosynthetic efficiency, in regulating carboxysome dynamics. Our data highlight a role for RcaE in perceiving external light cues and regulating carboxysome structure and function and, thus, in the cellular capacity for carbon fixation and organismal fitness.

## Abstract (precis)

Carboxysomes are proteinaceous subcellular compartments, or bacterial organelles, found in cyanobacteria that consist of a protein shell surrounding a core primarily composed of the enzyme ribulose-1,5-biphosphate carboxylase/oxygenase (RuBisCO) that is central to the carbon dioxide-concentrating mechanism (CCM) and carbon fixation. Whereas significant insights have been gained regarding the structure and synthesis of carboxysomes, limited attention has been given to how their size, abundance, and protein composition are regulated to ensure optimal carbon fixation in dynamic environments. Given the centrality of carboxysomes in photosynthesis, we provide an analysis of the role of a photoreceptor, RcaE, which functions in matching photosynthetic pigmentation to the external environment during complementary chromatic acclimation and thereby optimizing photosynthetic efficiency, in regulating carboxysome dynamics. Our data highlight a role for RcaE in perceiving external light cues and regulating carboxysome structure and function and, thus, in the cellular capacity for carbon fixation and organismal fitness.

## INTRODUCTION

Some cyanobacterial strains tune photosynthetic capacity to environmental cues, including changes in the availability of light. *Fremyella diplosiphon* is a filamentous, freshwater cyanobacterium that exhibits complementary chromatic acclimation (CCA), which is a process to primarily tune photosynthetic pigment type and levels to changes in the prevalent wavelengths of external light (1). In *F. diplosiphon*, CCA-associated changes occur in response to the presence and abundance of red versus green wavelengths of light (2). In red-enriched light, *F. diplosiphon* accumulates red-absorbing, green-colored phycocyanin in light-harvesting complexes to maximize light absorption for photosynthesis. Conversely, under green-enriched conditions, *F. diplosiphon* accumulates green-absorbing, red-colored phycoerythrin for promoting light harvesting. In addition to pigmentation changes, cell shape and filament length also are controlled by light during CCA (2). Cyanobacteriochrome (phytochrome-related) photoreceptor RcaE is known to control both the light-dependent regulation of pigmentation (3, 4) and cell and filament morphologies (5, 6), both of which are characteristic of CCA in *F. diplosiphon*. As wavelength-dependent tuning of pigmentation is linked to the maintenance of optimal photosynthetic efficiency (7), RcaE has a role in tuning photosynthetic potential to external light cues. In prior studies, we noted a reduction in the growth of a ∆*rcaE* mutant strain relative to the wild-type (WT) strain under conditions of ambient air in red and green light (8) and that expression of genes associated with inorganic carbon (Ci) uptake was generally upregulated in the ∆*rcaE* mutant relative to the WT (9). Together, these phenotypes suggest a high-carbon-requiring (HCR) phenotype associated with defects in biocarbonate uptake, with Ci uptake, or with some part of the CO_2_-concentrating mechanism (CCM).

Apart from suggesting a potential state of Ci deficiency in Δ*rcaE* cells, the impact of an absence of RcaE on the expression of Ci uptake genes is particularly significant as light has previously been reported to be required under low-Ci conditions for the expression of genes impacting inducible Ci uptake systems of the CCM in *Synechocystis* sp. strain PCC 6803 (here *Synechocystis*) (10, 11). Because light is required for this process, redox or phytochrome signals were implicated in the light-dependent cellular response to low Ci in cyanobacteria (12). The regulation of inorganic carbon uptake genes involved in the CCM in a Δ*rcaE* mutant provided genetic evidence of involvement of the photoreceptor RcaE in responses to low Ci (9).

The CCM is modular, with distinct components consisting of the Ci uptake systems at the membrane in addition to the intracellular carboxysome subcompartment (13–15). The carboxysome is a specialized protein-based bacterial microcompartment (BMC) containing ribulose-1,5-biphosphate carboxylase (RuBisCO), which functions in carbon fixation (16, 17). Although there have been significant insights into the structural makeup of carboxysomes (17) and the assembly principles of BMC shells (18), there have been limited insights about the environmental inputs that regulate the synthesis, positioning, and potential functional tuning of carboxysomes. Prior studies demonstrated that regular distribution and positioning of carboxysomes along the long axis of the cell are critical for maintaining carboxysome partitioning and associated cellular fitness during cell division (19). Notably, carboxysomes increase in number under low-carbon conditions in WT *Synechocystis* (20) and *Synechococcus* (21–23) strains. Additionally, increased light intensity leads to an increase in the transcription of carboxysome genes (24, 25) in *Synechocystis* sp. PCC 6803 and synthesis of carboxysomes (22) in *S. elongatus* PCC 7942. The increase in carboxysome number under elevated light conditions presumably increases the carbon fixation capacity as a coordinated and long-term acclimation response to an increase in photosynthetic potential under conditions of increased availability of photons to drive electron transport. In addition to the responses seen under conditions of high levels of light, the expression of carboxysome-related genes increases during the light cycle under diurnal conditions (26, 27) or during the subjective day under conditions of circadian growth (28). In one proteomic study performed with *Cyanothece*, carboxysome proteins also accumulated to higher levels in the light phase of a diel cycle (29). The mechanisms controlling environmental regulation of carboxysomes in cells have not received significant experimental attention.

Here, we report on an investigation of the regulation of cellular responses to dynamic light conditions, including coordinate regulation of light absorption capabilities and carboxysome number, structure, and function, in the CCA-capable species *F. diplosiphon*. Cells of a Δ*rcaE* mutant that is incapable of normal regulation of CCA exhibit smaller and apparently more numerous carboxysomes than WT cells. Thus, we assessed *ccm* gene expression and protein accumulation in the presence of the red and green wavelengths that are critical for CCA and in the WT strain compared to a Δ*rcaE* strain. Given the known phenotypes of altered cell shape and high accumulation of reactive oxygen species (ROS) in the Δ*rcaE* strain, we also assessed whether cell shape and intracellular ROS levels have an indirect impact on the carboxysome structure using cell shape mutants and ROS-mitigating compounds. Our results suggest a role for RcaE, including transcriptional regulation of *ccm* genes, in controlling carboxysome structure and number that may be linked to functional tuning of carboxysomes in response to external light cues.

## RESULTS

### RcaE regulates carboxysome size and abundance in *Fremyella diplosiphon.*

**FIG 1:**
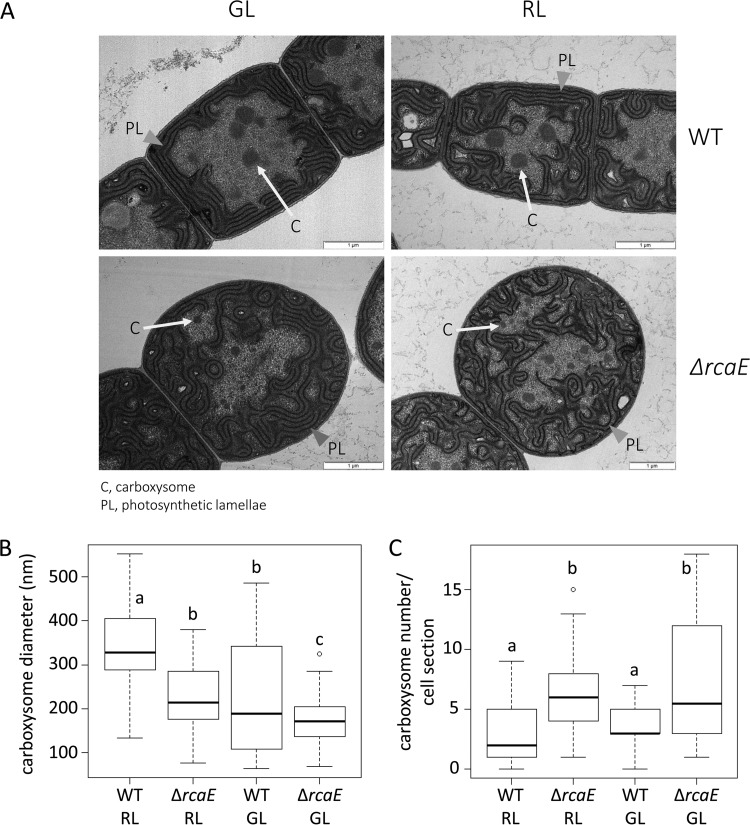
Carboxysome structure, size, and abundance determination in *Fremyella diplosiphon* strains under green (GL) and red (RL) light conditions. (A) Transmission electron microscopy (TEM) analysis of cellular morphology of *F. diplosiphon*. Representative images of SF33 wild-type (WT) pigmentation strain and ∆*rcaE* mutant strain under GL and RL are shown. C, carboxysomes (indicated by white arrows); PL, photosynthetic lamellae indicated by gray arrowheads. Bars, 1 µm. (B and C) Carboxysome size (B) and number per cell section (C) measurements of WT and ∆*rcaE* strains under GL and RL. To determine size, the maximum diameters of at least 25 carboxysomes from each strain were measured under each growth condition and are presented as a box plot. Box plots were used as they present the entire data population spread, ordered from smallest to largest. The horizontal bold line inside each box corresponds to the median, and the box covers the second and third quartile groups (the middle 50% of all values). The vertical line below the box corresponds to the first quartile group (the lowest 25% of all values), and the line above the box corresponds to the fourth quartile group (the highest 25% of all values). Presenting the entire spread of data allows visualization of differences between population spreads. Corresponding averages (± standard errors) can be found in Table 1. Statistical analyses were conducted using a Welch two-sample *t* test performed in R. Identical letters over bars represent homogenous mean groups (*P* > 0.05); different symbols indicate a statistically significant difference (*P* < 0.05) from others.

RcaE is known to control both light-dependent regulation of pigmentation (3, 4) and cell and filament morphologies (5) in *F. diplosiphon*. A RcaE-deficient strain of *F. diplosiphon* grows more slowly than the WT in ambient air (see Fig. SA1 in the supplemental material) (8) and has increased expression of Ci uptake genes (9); taken together, those two observations suggest that the Δ*rcaE* mutant has an HCR phenotype. To assess subcellular differences in the Δ*rcaE* mutant that may underlie such an HCR phenotype, we performed detailed ultrastructure analyses of WT and Δ*rcaE* strains grown under both RL and GL conditions using transmission electron microscopy (TEM). WT cells were more brick shaped and elongated under GL conditions than under RL conditions (Fig. 1A), and Δ*rcaE* cells were spherical in both RL and GL (Fig. 1A), as previously described for confocal laser scanning microscopy-based images (5). Photosynthetic lamellae (PL) were regularly arranged around the cell perimeter in WT cells grown under both RL and GL conditions (Fig. 1A). In contrast, PL were more irregularly arranged or dispersed in the Δ*rcaE* mutant cells under GL or RL conditions. Carboxysomes were larger in size in WT cells than in Δ*rcaE* mutant cells, independently of light conditions (Fig. 1A and B) (Table 1). Additionally, a comparison between RL and GL showed that carboxysomes were smaller under GL than under RL in both the WT and Δ*rcaE* strains. Although the carboxysome were smaller in size, the carboxysome number per cell section was significantly greater in Δ*rcaE* cells than in WT cells under both RL and GL conditions (Fig. 1C) (Table 1).

**TABLE 1:** Quantification of average carboxysome sizes and average numbers of carboxysomes per cell section

Parameter	Value(s) for indicated strain^a^									
	RL		GL		MRL		MGL		RL + AA	
	WT	Δ*rcaE*	WT	Δ*rcaE*	WT	Δ*rcaE*	WT	Δ*rcaE*	WT	Δ*rcaE*
Carboxysome size (nm)^b^	340 ± 19	224 ± 12*	227 ± 19#	174 ± 5* #	380 ± 22	317 ± 18* **	398 ± 25**	250 ± 13* ** #	328 ± 14	214 ± 11*
No. of carboxysomes/cell section	3.0 ± 0.3	6.2 ± 0.3*	3.8 ± 0.2	7.2 ± 0.3*	3.5 ± 0.2	7.1 ± 0.3*	2.8 ± 0.2**	6.7 ± 0.3*	3.5 ± 0.2	6.4 ± 0.2*
Sample size (*n*) for carboxysome size measurements	27	43	45	106	35	71	28	47	29	51
Sample size (*n*) for measurements of no. of carboxysomes/ cell section	91	186	114	215	105	178	28	47	35	64

^a^ Column headings indicate light conditions under which WT and Δ*rcaE* cells are grown. RL, red light at ~10 to 12 µmol·m^−2^·s^−1^; GL, green light at ~10 to 12 µmol·m^−2^·s^−1^; MRL, medium red light at ~30 µmol·m^−2^·s^−1^; MGL, medium green light at ~30 µmol·m^−2^·s^−1^; RL + AA, red light at ~10 to 12 µmol·m^−2^·s^−1^ with added ascorbic acid (AA) at 2 mM. Results of statistical analyses for *P* values of <0.05 are indicated as follows: *, WT strain versus Δ*rcaE* strain under the same conditions; #, GL versus RL for the same light intensity for the same strain; **, low light versus medium light for the same light quality for the same strain.

^b^ Numbers for carboxysome size and carboxysome/cell section are presented as averages ± standard errors.

### RcaE regulates carboxysome-associated gene expression and protein accumulation in *F. diplosiphon.*

**FIG 2:**
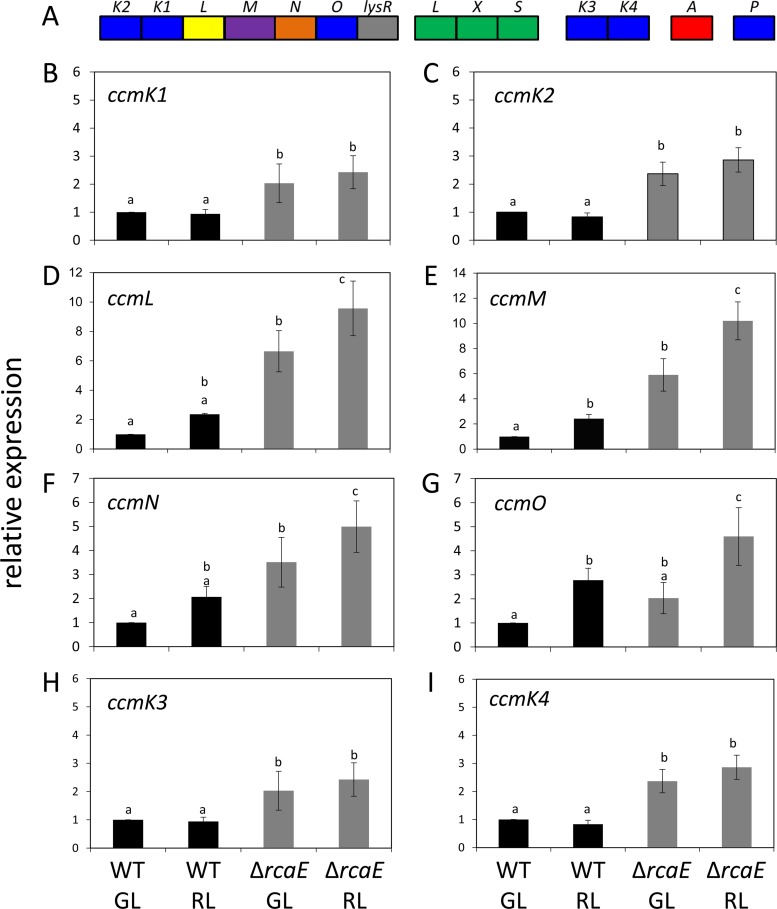
Carboxysome operons and quantitative PCR (qPCR)-based gene expression analyses in *Fremyella diplosiphon*. (A) Carboxysome-associated genes and operons found in *F. diplosiphon*. (B to I) Data represent levels of expression of *ccm* genes, including *ccmK1* (B), *ccmK2* (C), *ccmL* (D),* ccmM* (E), *ccmN* (F), *ccmO* (G), *ccmK3* (H), and *ccmK4* (I), in WT and Δ*rcaE* strains grown under green light (GL) or red light (RL). Levels of expression of genes are presented relative to the results determined for the internal control *orf10B*, and the data in each panel are shown relative to the expression level of the gene of interest in WT cells in GL. Bars represent averages (± SD) of data from three independent biological replicates. Identical letters over bars represent homogenous mean groups (*P* > 0.05); different symbols indicate a statistically significant difference (*P* < 0.05) from others.

Given the observed differences in carboxysome size, we assessed whether there were mutations in the sequences of known carboxysome genes by amplifying and sequencing target genomic regions. Similarly to those of other cyanobacteria containing beta carboxysomes, the key components of the carboxysome are encoded in a core *ccm* operon in *F. diplosiphon*, with other shell proteins encoded in disparate satellite locations in the genome (Fig. 2A). The core *ccm* operon encodes the shell proteins CcmK2, CcmK1, CcmL, and CcmO, as well as other components, including CcmN, which is essential for shell assembly, and CcmM, which facilitates RuBisCO nucleation (reviewed in reference 17). The essential CcmP is encoded at a separate genomic location (Fig. 2A). *F. diplosiphon* is one type of cyanobacterium that contains an expanded set of paralogs for proteins which comprise the carboxysome shell, including CcmK3 and CcmK4. These nonessential paralogs are often at disparate locations with respect to the core *ccm* operon and provide an expanded set of carboxysome shell subunits that have been hypothesized to afford selective advantages by altering carboxysome shell permeability (and thus function) under dynamic growth conditions (30, 31). Based on Sanger sequencing performed on PCR-amplified, *ccm* gene-containing regions of the genome, we identified no mutations in the sequences of known *ccm* or carboxysome genes (data not shown).

We proceeded to assess differences in the expression levels of *ccm* genes using data from a prior RNA-sequencing (RNA-seq) analysis comparing WT and Δ*rcaE* strains (32). All *ccm* genes, with the exception of *ccmO*, *ccmK3*, and *ccmP*, exhibited significantly increased mRNA levels in the Δ*rcaE* mutant relative to the WT in RNA-seq analysis (Table 2). Notably, *rbc* genes were largely downregulated in the Δ*rcaE* mutant compared to the WT (Table 2). We confirmed the differences for select *ccm* genes by quantitative reverse transcription-PCR (qRT-PCR) (Fig. 2B).

**TABLE 2:** RNA sequencing data for carboxysome genes from *F. diplosiphon* SF33 WT and Δ*rcaE* mutant strains grown under GL or RL conditions

Gene^a^	*Ava*^b^ homolog	No. of reads for indicated strain				Fold change for indicated strain			
						RL versus GL^c^		Δ*rcaE* versus WT^d^	
		WT		Δ*rcaE*		WT	Δ*rcaE*	GL	RL
		GL	RL	GL	RL				
*ccmK2*	Ava_4472	1,438.8	774.5	2,954.4	2,405.9	0.5*	0.81	2.05**	3.11**
*ccmK1*	Ava_4471	791.3	696	1,612.1	2,592.2	0.88	1.6*	2.04**	3.72**
*ccmL*	Ava_4470	362	390.7	776.1	1,335.9	1.08	1.7*	2.14**	3.42**
*ccmM*	Ava_4469	1,631.1	1,667.7	2,933.7	6,722.4	1.0	2.3**	1.80*	4.03**
*ccmN*	Ava_4468	1,061	1,409	2,073.1	3,137.2	1.3	1.5	1.95**	2.23*
*ccmO*	Ava_4467	951.2	6,310.3	639.4	6,767.9	6.6**	10.6**	0.67**	1.07
*lysR*	Ava_4466	366	397.4	344.2	451.6	1.1	1.3	0.94	1.14
									
*rbcL*	Ava_3907	11,568.3	13,244.9	8,839.7	29,880.3	1.1	3.4**	0.76*	2.26
*rbcX*	Ava_3906	4,522.2	5,419.3	1,248.8	2,586	1.2	2.1**	0.28**	0.48
*rbcS*	Ava_3905	3,828.1	6,486.9	1,390.1	3,087.2	1.7	2.2**	0.36**	0.48
									
*ccmK3*	Ava_4709	450.6	371.2	396.2	470.6	0.8	1.2	0.88	1.27
*ccmK4*	Ava_4710	329.4	152.6	519	427.7	0.46**	0.8	1.58	2.80**
									
*ccmP*	Ava_4911	32.2	36.5	47.5	33.8	1.13	0.7	1.48	0.93
*ccaA*	Ava_2165	70.4	61.4	51	177.5	0.87	3.5**	0.72*	2.89**

^a^ The gene grouping indicates which genes are near each other in the genome (*ccmP* and *ccaA* are isolated from the others in distinct regions of the genome for each).

^b^ ORFs were compared against *Anabaena* *variabilis* (Ava) ATCC 29413 annotated proteins using BlastX with a cutoff E value of 0.0001 to determine Ava homolog.

^c^ Fold change data were calculated for each strain by differential expression analysis of comparisons of the results determined for two light treatments. *, *P* < 0.5; **, *P* < 0.01 (significance value calculated for RL counts versus GL counts for each strain).

^d^ Fold change data were calculated for each light condition by differential expression analysis of comparisons of the results determined for the two strains. *, *P* < 0.5; **, *P* < 0.01 (significance value calculated for the Δ*rcaE* strain counts versus WT strain counts for each light condition).

**FIG 3:**
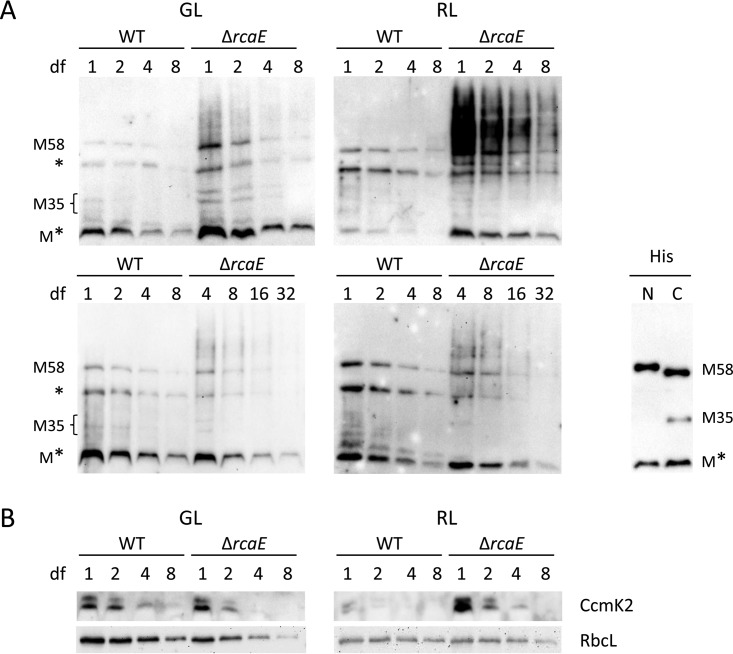
Immunoblot analyses of carboxysome protein accumulation in *Fremyella diplosiphon*. Ccm protein accumulation in the SF33 WT and Δ*rcaE* strains under green (GL) and red (RL) light conditions is shown in representative blots. Protein extracts (measured in micrograms of undiluted total protein extract [indicated in parentheses]) and 2-fold dilutions as indicated by dilution factors (df; numbers above lanes) were loaded for assessment of CcmM (75 μg) (A) and of CcmK2 (75 μg) and RbcL (20 μg) (B). After blotting was performed, proteins were detected using anti-CcmM (1:5,000 dilution, 3-min exposure), anti-CcmK2 (1:3,000 dilution, 1-min exposure), or anti-RbcL (1:20,000 dilution, 4-min exposure) antibodies. For panel A, distinct CcmM variants are indicated, which include *F. diplosiphon* versions of full-length CcmM-58 (M58), Ccm-35 (M35) (derived from an internal ribosome entry site) (33) and a reproducibly observed ~30-kDa band that we designated M* and which is also observed in *E. coli*-expressed, N-terminal or C-terminal His-tagged expressed versions of CcmM purified via Ni-NTA affinity chromatography (panel A, lower right). M* protein was sequenced and found to contain peptides which map to regions throughout the full-length Ccm protein (see Fig. SA2). *, nonspecific band detected with the anti-CcmM antibodies. The lower portion of panel A with increased dilutions of soluble protein from the Δ*rcaE* strain was included to allow comparison of the WT and Δ*rcaE* strains in a range of protein levels that were not saturating for the Δ*rcaE* strain, given its significantly higher accumulation of CcmM-reactive bands in the same dilution range as that shown in upper portion of the panel.

To assess whether the observed transcriptional responses were also apparent at the protein level, CcmM, CcmK2, and RbcL proteins were examined using immunoblot analyses. CcmM and CcmK2 proteins accumulated to higher levels in the Δ*rcaE* strain than in the WT strain (Fig. 3), reflecting that these factors are regulated at the transcriptional level. CcmM exhibits two forms in cells, due to the presence of an internal ribosome binding site on the transcript (33). This results in two distinct forms of CcmM, i.e., an ~58-kDa form (CcmM-58 or M-58) and an ~35-kDa form (CcmM-35 or M-35), that accumulate in cyanobacteria. CcmM-58 levels were especially elevated in the Δ*rcaE* strain relative to the WT, although CcmM-35 levels were somewhat elevated, particularly in GL (Fig. 3A). Thus, the ratio of CcmM-58 to CcmM-35, in addition to total CcmM levels, changed in the WT strain relative to the Δ*rcaE* mutant. Prior analyses in which the ratio of M-58 to M-35 was altered by overexpressing the full-length CcmM or CcmM-35 version resulted in an alteration in carboxysome size in *Synechococcus* sp. PCC 7942 (33). In those studies, a reduction in CcmM-58 relative to CcmM-35 levels resulted in larger carboxysomes, whereas increased CcmM-58 levels relative to Ccm-35 levels were correlated with smaller carboxysomes (33). Notably, another band at approximately 30 kDa was detected by the anti-CcmM antibody (i.e., M* in Fig. 3A) and accumulated to higher levels in the Δ*rcaE* mutant than in the WT. We conducted protein sequence analysis to determine the identity of this band and determined that peptides for this protein map throughout the full-length CcmM sequence (Fig. SA2), indicating overaccumulation of CcmM-derived bands in the Δ*rcaE* strain.

As expected on the basis of transcriptional downregulation in GL, RbcL levels were significantly lower in the Δ*rcaE* mutant under these conditions (Fig. 3B). Based on densitometry analysis, RbcL levels were reduced by 64% (standard deviation [SD] = 0.07, *n* = 5) in the Δ*rcaE* strain compared to the WT under GL conditions. Under RL conditions, RbcL protein levels may either decrease slightly or remain roughly constant, in contrast to the observed transcriptional upregulation. We observed a 31% reduction (SD = 0.31, *n* = 6) in RbcL levels in the Δ*rcaE* strain compared to the WT in RL. Together, these findings suggest an overaccumulation of carboxysome shell proteins and of the CcmM protein which functions in nucleating the cargo relative to the levels of the carboxysome RuBisCO cargo, which were lower in the Δ*rcaE* mutant than in the WT.

### RcaE regulates the response of carboxysome structure to changes in light quality and intensity in *Fremyella diplosiphon.*

Due to the ability of photoreceptors such as RcaE to respond to light quantity in addition to light quality (34–36) and the prior correlation of increased carboxysome numbers under conditions of increased light intensity in a cyanobacterium (22), we assessed carboxysome structures in WT and Δ*rcaE* strains under a variety of light conditions. Using TEM-based analyses, we measured carboxysome diameter and number/cell section for both strains grown at ~30 µmol·m^−2^·s^−1^ in medium RL (MRL) or GL (MGL) conditions. The Δ*rcaE* strain retained a small-carboxysome phenotype relative to the WT under both MRL and MGL conditions (Table 1). However, the higher light intensity resulted in statistically significantly larger carboxysomes in the Δ*rcaE* strain under both MRL and MGL conditions, as well as in the WT under MGL conditions, than under standard (i.e., 10 to 15 µmol·m^−2^·s^−1^) light conditions (Table 1). However, no difference in size was noted in comparisons of WT cells grown under MGL conditions to those grown under MRL conditions or of WT cells grown under MRL conditions to those grown under standard RL conditions (Table 1). Thus, the loss of RcaE leads to light-dependent changes under all conditions, even if no changes are observed in the WT strain.

### RcaF and RcaC do not function with RcaE in the regulation of carboxysome-associated gene expression in *F. diplosiphon.*

**FIG 4:**
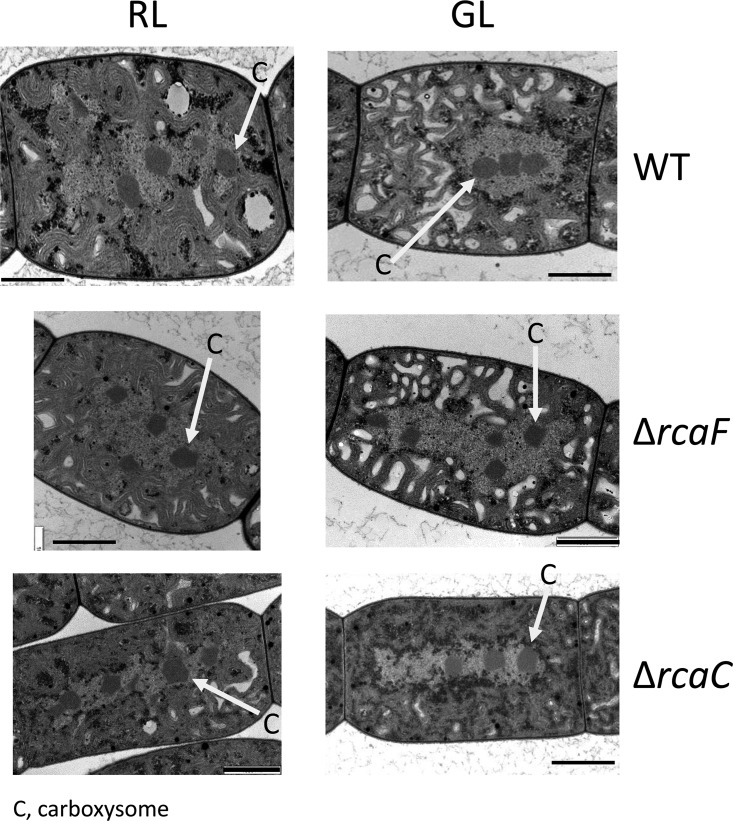
Transmission electron microscopy (TEM) analysis of carboxysome morphology of *Fremyella diplosiphon* strains under green (GL) and red (RL) light conditions. Representative images of the SF33 wild-type (WT) pigmentation strain (top), ∆*rcaF* mutant strain (middle), and ∆*rcaC* mutant strain (bottom) are shown. C, carboxysomes (indicated by white arrows). Bar, 1 µm.

RcaF and RcaC function downstream of RcaE in the regulation of pigmentation (37). Although they are not required for RcaE-dependent regulation of morphology in GL, RcaF and RcaC contribute to morphology regulation under RL conditions (38). To determine whether these effectors function downstream of RcaE in the regulation of carboxysome structure in cells, we assessed carboxysomes in Δ*rcaF* and Δ*rcaC* mutants. However, the carboxysomes in these mutants were very similar in appearance to those in WT cells (Fig. 4). Thus, RcaE-dependent regulation of carboxysomes does not occur through known response regulators RcaF or RcaC, as carboxysomes in either Δ*rcaF* or Δ*rcaC* mutants do not differ significantly from those in the WT strain. In additional support of this TEM-based observation, the expression of *ccm* genes was not altered significantly in either the Δ*rcaF* strain or the Δ*rcaC* strain (Fig. SA3). Thus, RcaE appears to function primarily through the activity of unknown effectors to regulate carboxysome structure.

### RcaE-dependent regulation of cell shape and intracellular ROS levels is not correlated with the regulation of carboxysome structure in *F. diplosiphon.*

**FIG 5:**
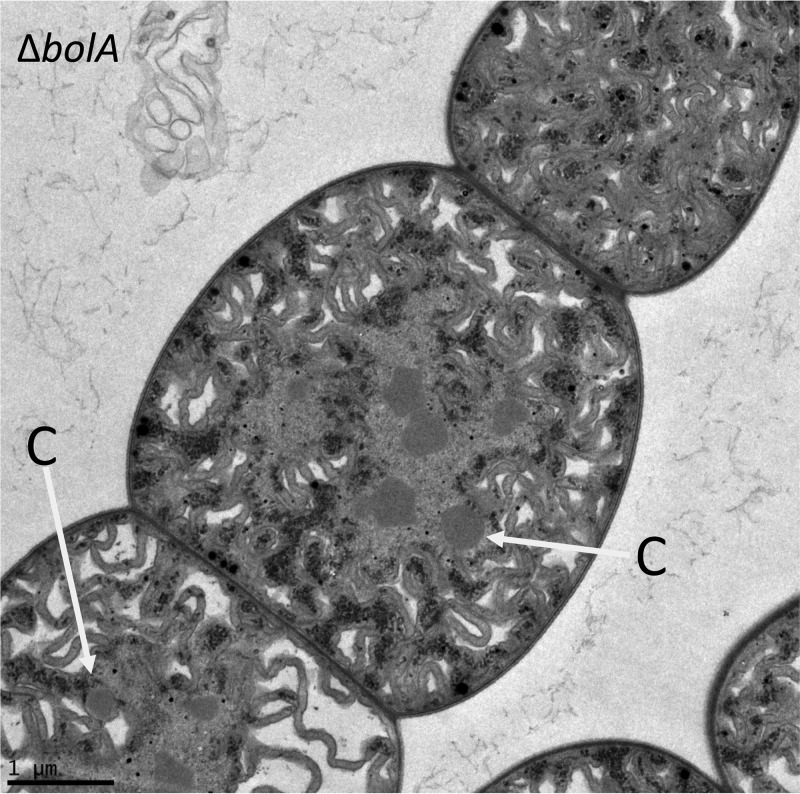
Transmission electron microscopy (TEM) analysis of the ultrastructure of the Δ*bolA* strain of *Fremyella diplosiphon*. One representative image is shown. C, carboxysomes (indicated by white arrows). Bar, 1 µm.

Initial assays indicated that, in addition to being smaller and more numerous in the Δ*rcaE* strain than in the WT, carboxysomes were occasionally mislocalized among thylakoid membranes rather than exhibiting the expected location in the cytosol in Δ*rcaE* mutant cells (Fig. 1A). Prior studies indicated movement of carboxysomes from the central cytoplasm to the cell periphery under conditions of low inorganic carbon levels (23). To determine whether this mislocalization phenotype or the observed carboxysome structural defect phenotype was primarily correlated with the spherical cell shape of the Δ*rcaE* mutant or with other parameters, we assessed another spherical mutant of *F. diplosiphon*, i.e., the Δ*bolA* mutant (6). BolA is a morphogene that was shown previously to be involved in regulation of cell shape in a number of bacteria (39, 40). The deletion of *bolA* is associated with large spherical cell shape in a Δ*bolA* mutant of *F. diplosiphon* (6), and its overexpression induces a spherical cell morphology (41). The Δ*bolA* mutant exhibited WT-sized carboxysomes, and yet these structures were occasionally mislocalized and found closer to the periphery of the thylakoid membranes, rather than centrally in the cytosol, in cells (Fig. 5).

**FIG 6:**
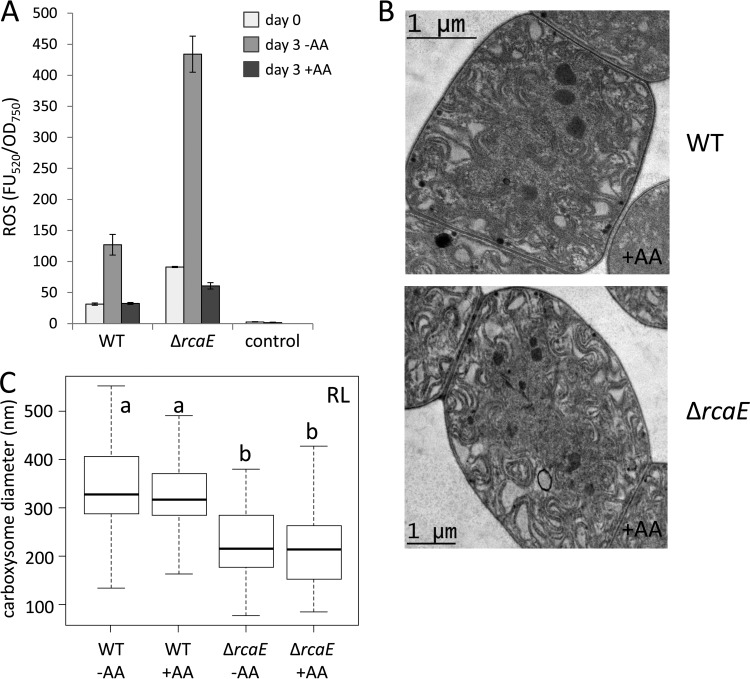
Accumulation of reactive oxygen species (ROS) and carboxysome structure in *Fremyella diplosiphon*. (A) ROS-dependent DCF fluorescence and cell component fluorescence in *F. diplosiphon* under RL after 3 days of treatment with ascorbic acid (2 mM) (+AA) or without ascorbic acid (-AA) added to the growth medium in SF33 and Δ*rcaE* strains. The control represents BG-11 medium plus DCFH-DA fluorescence without cells. Bars represent the average levels of fluorescence (measured as fluorescent units [FU] expressed in arbitrary values) at 520 nm relative to optical density (OD) at 750 nm (FU_520_/OD_750_). Error bars indicate SD. (B) Transmission electron microscopy (TEM) analysis of strains under RL grown in the presence of AA. Bars, 1 µm. (C) Carboxysome size measurements of WT and ∆*rcaE* strains under RL, with and without AA. To determine size, the maximum diameters of at least 25 carboxysomes from each strain were measured under each growth condition and are presented as a box plot, with the bold line signifying the median diameter, the box representing the second and third quartile groups (the middle 50% of all values), and the lower vertical line corresponding to the first quartile group (the lowest 25% of all values) and the upper line corresponding to the fourth quartile group (the highest 25% of all values). Corresponding averages (± standard errors) can be found in Table 1. Statistical analyses were conducted using a Welch two-sample *t* test performed in R. Identical letters over bars represent homogenous mean groups (*P* > 0.05); different symbols indicate a statistically significant difference (*P* < 0.05) from others.

Given the prior recognition that the redox state of the cell may impact CcmM activity (42), we investigated whether the high ROS levels characteristic of Δ*rcaE* cells might contribute to the observed disruptions in CcmM levels and carboxysome phenotypes observed in this strain. As previously reported, Δ*rcaE* cells accumulated elevated levels of reactive oxygen species (ROS) (Fig. 6A) (43, 44). Thus, we investigated whether intracellular ROS accumulation is correlated with the smaller carboxysomes apparent in the Δ*rcaE* strain. To investigate the potential role of ROS in regulating carboxysome size, we treated Δ*rcaE* and WT cells with the ROS-mitigating antioxidant ascorbic acid (AA) (44). AA-treated Δ*rcaE* cells exhibited reduced intracellular ROS levels compared to the untreated parental Δ*rcaE* strain (Fig. 6A) (44). However, there were no significant differences between the sizes of carboxysomes in Δ*rcaE* cells in the presence or absence of AA, and the carboxysomes were significantly smaller than those seen with the WT controls in each case (Fig. 6B and C). Furthermore, the aforementioned Δ*bolA* strain also has elevated intracellular ROS levels (6, 41), and the levels were not correlated with a change in carboxysome size in this strain relative to the WT (Fig. 5).

### The structures of polyphosphate bodies are also regulated by RcaE in *F. diplosiphon.*

**FIG 7:**
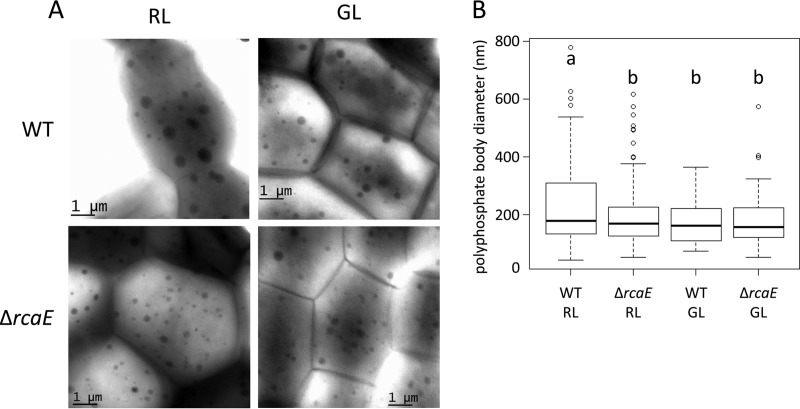
Assessment of polyphosphate body (PPB) structure via transmission electron microscopy (TEM) of whole cells of *Fremyella diplosiphon* strains. (A) Representative TEM images of the SF33 wild-type (WT) strain and ∆*rcaE* mutant strain grown under RL and GL are shown. Dark spots represent electron-dense bodies. (B) Quantification of diameters of polyphosphate bodies. To determine size, the diameters of at least 25 PPB from each strain were measured under each growth condition and are presented as a box plot, with the bold line signifying the median diameter, the box representing the second and third quartile groups (the middle 50% of all values), the lower vertical line corresponding to the first quartile group (the lowest 25% of all values), and the upper line corresponding to the fourth quartile group (the highest 25% of all values). Statistical analyses were conducted using a Welch two-sample *t* test performed in R. Identical letters over bars represent homogenous mean groups (*P* > 0.05); different symbols indicate a statistically significant difference (*P* < 0.05) from others.

To independently assess whether the smaller carboxysomes and the greater numbers of carboxysomes per cell section of a Δ*rcaE* mutant observed in thin-section TEM analysis represent a smaller size and yet a larger number of total carboxysomes in whole cells or an alteration in total carboxysome volume, we attempted to assess the whole-cell population of carboxysomes. We used negative staining of whole cyanobacterial cells with TEM analysis (45). Results from these analyses indicated a larger number of smaller electron-dense bodies that appeared to have the potential shape of carboxysomes in Δ*rcaE* cells than in the WT cells, especially in RL (Fig. 7). To confirm the identity of these structures, we used negative whole-cell staining of *Synechococcus* WT cells and a carboxysome-deficient strain as potential controls (Fig. SA4). Upon observing similar electron-dense bodies in those two lines, we conducted combined scanning TEM (STEM) and energy-dispersive X-ray spectroscopy (EDX) elemental analyses to identify the smaller, more numerous structures apparent in Δ*rcaE* cells. EDX analyses indicated that the bodies observed were polyphosphate bodies (PPB) (Fig. SA5). This finding indicated an unexpected role for RcaE in regulating both carboxysome and polyphosphate body (PPB) size and abundance.

In bacteria, *ppk* (encoding polyphosphate kinase 1) and *ppx* (encoding exopolyphosphatase) are involved in synthesis and degradation of polyphosphate (reviewed in reference 46). Notably, in negative-staining TEM analysis, a Δ*ppk* mutant in *Synechococcus* lacked dark bodies similar to those we observed in *F. diplosiphon* (47). Additionally, the Δ*ppk* mutant had altered regulation of several *ccm* genes compared to the WT, indicating a potential functional correlation between disruptions in PPB formation and carboxysome synthesis (47). Given these observations, we assessed whether *ppk* and *ppx* mRNA levels were altered in our RNA-seq data. However, there were no significant differences in the mRNA levels of these genes accumulating in the Δ*rcaE* mutant versus the WT (see Table SA1 in the supplemental material). Thus, RcaE appears to regulate carboxysomes through transcriptional control of carboxysome genes, and yet the disruption in PPB in the Δ*rcaE* mutant occurs without significant regulation of expression of genes known to impact PPB formation.

### Total carboxysome population size or volume is regulated by RcaE in *F. diplosiphon.*

As an alternative to negative-stain TEM analysis to determine whether the smaller carboxysomes of the Δ*rcaE* mutant represent a smaller size of and yet a larger total population of carboxysomes in cells, we counted the number of carboxysomes observed in a series of TEM thin sections and estimated the total number per cell on the basis of prior methods (48). These analyses indicated that the Δ*rcaE* mutant did indeed have a larger number of smaller carboxysomes per cell section than the WT strain under both GL and RL conditions (Fig. 1C) (Table 1).

Given the correlation between changes in light intensity and changes in carboxysome size, we also assessed whether the number of carboxysomes increased under conditions of increased light intensity. Light intensity typically did not alter the average carboxysome number per cell in either strain, except for a slight decrease in the WT strain under MGL conditions (Table 1). Similarly, there were no significant differences in carboxysome number seen in comparisons of standard RL and GL in either strain. This suggests that the number of carboxysomes per cell is well maintained in the WT strain, that the number is dependent on the presence of RcaE in *F. diplosiphon*, and that carboxysome size is primarily sensitive to dynamic photoenvironments.

## DISCUSSION

Here, we report a regulatory role for RcaE in maintaining carboxysome size and quantity per cell and in contributing to carboxysome subcellular localization in *F. diplosiphon*. The Δ*rcaE* mutant, which lacks the cyanobacteriochrome RcaE photoreceptor (3), has smaller and more numerous carboxysomes than the parental WT line. These observations provide evidence that RcaE contributes to the regulation of carboxysome size and quantity in *F. diplosiphon*. Carboxysomes are also mislocalized occasionally within the thylakoid membranes of this strain. Notably, prior studies have reported a shift in the location of carboxysomes from the central cytoplasm to the periphery of the cell under conditions of reduced inorganic carbon availability (23).

Polyphosphate body (PPB) morphology was also disrupted in the Δ*rcaE* strain, with more numerous and smaller PPB than were observed for WT cells. This is notable given several prior recognized associations between phosphate-rich PPB and carboxysomes in bacteria. In one proteobacterium study, the position of PPB correlated with the positioning and structure of carboxysomes (49). More closely related to the work here, carboxysomes have been previously reported to be closely associated or grouped with PPB in some cyanobacterial strains (50, 51). Whether these associations point to functional interaction remains to be definitively determined; however, misregulation of *ccm* genes in a *Synechococcus* mutant lacking PPB hints at a functional association (47). Given the noted association of PPB with DNA in the cytoplasm (52, 53, 74), the physical colocalization may indicate that carboxysomes are also nearby or associated with DNA. This association allows a number of possible connections between chromosome condensation/decondensation dynamics, gene expression regulation, and subcellular structures to be explored, especially since the Δ*rcaE* mutant exhibits disruptions to both carboxysome and PPB morphology. Our observations that only some *ccm* genes were significantly misregulated, that *ccm* genes in disparate regions of the genome were misregulated, and that there was no apparent change in expression of the *ppk* and *ppx* genes which are associated with PPB synthesis in the Δ*rcaE* mutant suggest that disruptions to carboxysome and PPB structures do not arise from nonspecific changes to chromosome accessibility in the nucleoplasm. However, the shared structural phenotypes of carboxysomes and PPB, alongside their previously reported associations, most likely highlight robust (and perhaps functional) interconnectivity between these subcellular structures. Taking the results together, we hypothesize that RcaE could have a role in multiple aspects of carboxysome regulation, including interactions with PPB, that are likely critical for carboxysome dynamics and function in carbon fixation in a cyanobacterium.

RcaE was previously described as the photosensory receptor that controls pigmentation and cellular morphology in *F. diplosiphon* (3–5). RcaE works through the activity of two known response regulators, RcaF and DNA-binding transcriptional regulator RcaC, in regulating pigmentation (3, 37, 54–59) and red-light-dependent regulation of cellular morphology (38). Notably, however, RcaE does not appear to function through RcaF and RcaC in the regulation of carboxysomes, as Δ*rcaF* and Δ*rcaC* mutants have no apparent defects in the regulation of carboxysome size or positioning and exhibit no significant misregulation of expression of major *ccm* genes *ccmM* and *ccmK2*. In the regulation of cellular morphology, RcaE controls expression of the *bolA* morphogene (6, 41). And yet RcaE also does not impact carboxysome morphology via BolA regulation, as a Δ*bolA* mutant has WT-like carboxysomes. Thus, although RcaE impacts expression of carboxysome genes and carboxysome structure and number, the effectors through which it functions to do so appear to be independent of known RcaE-regulated effectors controlling pigmentation and cell shape phenotypes characteristic of CCA.

Of note, localization of carboxysomes may be correlated with cell shape generally, as carboxysomes are mislocalized to the periphery of cells in mutants with a constitutive spherical morphology, including both the Δ*bolA* mutant and the Δ*rcaE* strain. Previously, additional correlations between carboxysomes and cell shape were made. Elongated cell division mutants exhibit decreased carboxysome numbers per cell and carboxysome structural defects (60). Notably, these mutants also have reduced levels of carboxysome-associated proteins (61). Additionally, impairments in cell morphology due to cytoskeleton defects were correlated with altered spatial distribution or mislocalization of carboxysomes in cells (19).

In addition to its spherical morphology, the Δ*rcaE* mutant has elevated intracellular ROS levels (43, 44). Despite both Δ*rcaE* and Δ*bolA* strains having elevated ROS levels (6, 44), Δ*rcaE* mutant cells have smaller carboxysomes and Δ*bolA* mutant cells have WT-sized carboxysomes. Additionally, even when intracellular ROS levels were reduced in Δ*rcaE* mutant cells treated with an antioxidant, carboxysomes were smaller in cells lacking RcaE. Thus, RcaE appears to have a direct regulatory role in controlling carboxysome morphology and dynamics, rather than indirectly impacting carboxysomes through altering intracellular ROS accumulation.

The regulatory role of RcaE related to carboxysome structure and function is linked to transcriptional regulation of *ccm* and carboxysome-associated genes. Ccm structural proteins overaccumulate and carboxysome cargo protein RuBisCO underaccumulates in a Δ*rcaE* mutant relative to the levels seen in the WT strain. This observed shift in the carboxysome protein profile results in reduced cargo and simultaneously elevated levels of the shell and CcmM-58, which may contribute to the generation of smaller, more numerous carboxysomes.

*F. diplosiphon* is able to adjust carboxysome size in response to a number of changes in its photoenvironment. Carboxysomes in the WT strain respond to increased light availability through an increase in size, with this effect being more pronounced under GL conditions. Carboxysomes also appear to be larger under red light than under green light at low light levels, but this effect is lost at higher light intensities. These data are consistent with a higher level of linear electron flow driving a larger need for carbon fixation. Since these general behaviors are not entirely lost in the Δ*rcaE* strain, more cellular factors are implicated in the light-dependent regulation of carboxysome structures. However, the loss of RcaE severely limited the maximum size of carboxysomes while increasing their number under all light conditions studied. Moreover, a larger number of light-dependent differences in carboxysomes were observed in the Δ*rcaE* strain whereas the WT strain showed limited light dependence, suggesting that RcaE is required to maintain carboxysome homeostasis in dynamic photoenvironments. This tendency to regulate carboxysome structure encourages future analyses of the extent to which RcaE-dependent alterations to carboxysome size and distribution can specifically impact carbon fixation.

Together, these results suggest that RcaE has a critical role in regulating carboxysome structure which likely serves to match carbon fixation potential with external light cues. Given the light-dependent regulation of expression of *ccm* genes in RL versus GL and the altered levels of expression of *ccm* and carboxysome-associated genes in the RcaE-deficient strain relative to the WT, both the structure (i.e., size and quantity) and composition (e.g., elevated *ccmL*, *ccmM*, *ccmO*, and *ccmN* expression in RL versus GL and reduced levels of RbcL in GL versus RL) of carboxysomes appear to be regulated and, indeed, fine-tuned in response to external light cues. Such a role for RcaE provides a key mechanism for matching the carbon-fixation capacity and photosynthetic potential of cells to available light. Given the prior observations that light intensity also regulates carboxysome structure and dynamics (22, 24, 25) and that phytochrome-related photoreceptors respond to light intensity in addition to light quality (62–64), we propose that RcaE plays a central role in tuning the structure and function of carboxysomes in response to a dynamic photoenvironment to optimize organismal fitness in *F. diplosiphon*.

## MATERIALS AND METHODS

### Supplemental text.

Supplemental text for this article is provided in Text S1 in the supplemental material.

10.1128/mSphere.00617-17.1 TEXT S1  Supplemental text. Download TEXT S1, PDF file, 0.1 MB. Copyright © 2018 Rohnke et al. 2018 Rohnke et al. This content is distributed under the terms of the Creative Commons Attribution 4.0 International license.

### Culture conditions.

Two strains of *Fremyella diplosiphon* were compared in this study: a short-filament wild-type pigment strain (here WT), which was identified as SF33 (65), and a RcaE-deficient (Δ*rcaE*) mutant strain (3). Strains were grown in BG-11 medium (Fluka, Buchs, Switzerland) with 20 mM HEPES (pH 8.0) (here referred to as BG-11/HEPES) at 28°C with continuous shaking at 175 rpm under continuous light conditions. Liquid starter cultures were inoculated from strains maintained on solidified BG-11/HEPES media (BG-11/HEPES containing 1.5% [wt/vol] agar) and grown under conditions of continuous white light from fluorescent light tubes (General Electric; model no. F20T12/PL/AQ/WS) at ~15 µmol·m^−2^·s^−1^. Exponentially growing cultures were diluted to an initial optical density at 750 nm (OD_750_) of ~0.05 and were transferred to the experimental culture conditions as indicated. Absorbance measurements were made with a SpectraMax M2 spectrophotometer (Molecular Devices, Sunnyvale, CA).

Red light (RL) and green light (GL) conditions were obtained using monochromatic growth chambers at an intensity of ~10 to 12 µmol·m^−2^·s^−1^ continuous broad-band RL (CVG sleeved Rosco red 24 fluorescent tubes) (General Electric; model no. F20T12/R24) or continuous broad-band GL (CVG sleeved Rosco green 89 fluorescent tubes) (General Electric; model no. 20T12/G78) as previously described (5). Growth under medium light intensity utilized ~30 µmol·m^−2^·s^−1^ continuous RL (model no. 2506RD light-emitting-diode [LED] grow light; LED Wholesalers Inc., Hayward, CA) (λmax, 660 nm) or GL (Sunbow model no. Sn 1320001-004; Geneva Scientific LLC) (λmax, 530 nm). For cultures grown to test supplemental carbon dioxide conditions, we used white light (WL) conditions in a Percival I-41LL incubator equipped with Phillips Alto II fluorescent lights (model no. F17T81TL841) under either air or 3% CO_2_. Light intensities were measured with a LI-250 light meter (Li-COR, Lincoln, NE) equipped with a quantum sensor (model LI-190SA).

### Transmission electron microscopy (TEM) and energy-dispersive X-ray (EDX) analysis. (i) TEM analysis of sectioned cells.

For conventional TEM, an ~10-ml volume of cells was harvested from exponentially growing cultures (i.e., OD_750_ at 0.6 to 0.8) by centrifugation at 5,125 × *g* at room temperature for 6 min. Spent medium was decanted, and pellets were resuspended in the remaining medium (~200 µl) and transferred to 1.5-ml microcentrifuge tubes. Cells were then centrifuged at 16,000 × *g* at room temperature for 5 min, and the remaining medium was removed. Cells were prefixed via resuspension with 1 ml of 2.5% (wt/vol) glutaraldehyde–2.5% (wt/vol) paraformaldehyde–0.1 M cacodylate buffer and incubated for 5 min at 33°C and 35% power in a Precision Pulsed Laboratory 9000 microwave oven (Electron Microscopy Sciences, Hatfield, PA). After three 10-min washes with 1 ml of 0.1 M cacodylate buffer, the cell pellets were resuspended in 2% (wt/vol) molten agarose in double-distilled water (dH_2_O) and then centrifuged for 1 min. The solidified agarose plug was removed from the microcentrifuge tube, and the dense, embedded cell pellet was cut into cubes (~1 to 4 mm^3^). Embedded cells were washed three times for 10 min each time with cacodylate buffer and then postfixed with 2% (wt/vol) osmium tetroxide–cacodylate buffer for 5 min and heated in the microwave oven at 33°C and 35% power. Cells were then washed three times for 10 min each time using 0.1 M cacodylate buffer followed by three 10-min washes using dH_2_O. Postfixed cells were blocked with 2% (wt/vol) uranyl acetate (Electron Microscopy Sciences, Hatfield, PA)–dH_2_O and heated in the microwave oven for 5 min at 33°C and 35% power, which has been reported to enhance contrast for carboxysomes (66). Following three 15-min washes with dH_2_O, fixed cells were dehydrated in an acetone series (30%, 50%, 70%, 80%, 90%, 95%, 100%, 100%, and 100%) in 20-min intervals either using an EMP5160 tissue processor (Boeckeler Instruments, Inc., Tucson, AZ) or manually. Dehydrated samples were infiltrated with Spurr resin (Firm Standard; Electron Microscopy Sciences, Hatfield, PA) in a 3:1, 2:2, and 1:3 series of acetone/Spurr resin for 2 to 3 h at room temperature or overnight at 4°C at each step. Infiltrated cells were soaked in Spurr resin for 48 h with 3 exchanges of resin, and then blocks were cured at 60°C for 48 h. Thin sections were prepared using a PowerTome XL ultramicrotome (Boeckeler Instruments, Inc., Tucson, AZ), and 70- to 90-nm-thick sections (estimated from silver to gold interference color) were placed on 200-mesh Cu grids (Electron Microscopy Sciences, Hatfield, PA). Grids were stained with 4% (wt/vol) osmium tetroxide–dH_2_O for 30 min followed by Reynold’s formula (lead citrate, comprising lead nitrate and sodium acetate; Electron Microscopy Sciences, Hatfield, PA) for 15 min while covered alongside NaOH pellets. Sections were imaged using a Jeol 100CX TEM (Jeol USA Inc., Peabody, MA) equipped with a MegaViewIII digital camera at an operating voltage of 100 V.

### (ii) Negative-staining TEM analysis of whole cells.

For negative-staining TEM analysis of whole cells, 2 ml of cells at an OD_750_ of ≥0.2 or 5 ml of cells at an OD_750_ of ≤0.2 was harvested by centrifugation at 5,125 × *g* at room temperature for 6 min. Spent medium was decanted, pellets were resuspended in 15 ml of dH_2_O, and then cells were centrifuged at 5,125 × *g* at room temperature for 6 min. The supernatant was discarded by aspiration, and the pellets were resuspended in 0.5 ml dH_2_O. A 5-µl aliquot of resuspended cell pellet was placed on a 200-mesh Cu grid coated with Formvar (Electron Microscopy Sciences, Hatfield, PA) and incubated for 2 min at room temperature. The grid was blotted nearly dry with Whatman filter paper. Either 5 µl of 0.1% (wt/vol) uranyl acetate–dH_2_O (stained condition) or 5 µl of dH_2_O (unstained condition) was added to the grid and blotted away after 5 s. Grids were then washed once with 5 µl of dH_2_O for 5 s and then blotted nearly dry. Grids were imaged using a Jeol 100CX TEM equipped with a MegaViewIII digital camera at an operating voltage of 100 V.

### Carboxysome and polyphosphate body size and number quantification.

To determine sizes of carboxysomes and polyphosphate bodies (PPB), the diameters of at least 25 of each from each strain were measured in TEM images under each growth condition. Analysis was done in the image editing software Paint.net, and we selected the maximum diameter for consistency in representing irregular shapes. The numbers of carboxysomes and PPB were determined by counting positively identified structures in ~30 cell sections using TEM sections (for carboxysomes) or ~10 cells using negative-staining TEM (for PPB) for each strain under each condition. Positive identification of a carboxysome structure (for both size and number) satisfied three criteria: (i) appearance of some sharp edges; (ii) moderate electron density in contrast to the cytosol; (iii) regular, paracrystalline distribution of electron densities within the carboxysome. The negative-staining technique highlighted cell outlines and allowed visualization of the naturally electron-dense PPB. For quantification of both carboxysomes and polyphosphates, we used box plots to display data. Box plots were used as they present the entire data population spread, ordered from smallest to largest. The horizontal bold line inside each box plot graph corresponds to the median, and the box covers the second and third quartile groups (the middle 50% of all values). The vertical line below the box corresponds to the first quartile group (the lowest 25% of all values) and the line above the box corresponds to the fourth quartile group (the highest 25% of all values). Presenting the entire spread of data allows visualization of differences between population spreads. Averages (± standard errors) are also presented for carboxysome size and number in tabulated format.

### Quantitative RT-PCR analyses.

The abundance of *ccmK1*, *ccmK2*, *ccmL*, *ccmM*, *ccmN*, *ccmO*, *ccmK3*, and *ccmK4* transcripts in total RNA extracted from GL- and RL-grown WT or ∆*rcaE* strains of *F. diplosiphon* was analyzed using the delta delta threshold cycle (ΔΔ*C*_*T*_) method as detailed previously (6). In brief, total RNA was extracted as described previously (67, 68) and reverse transcribed (0.5 μg in a 20-μl reaction mixture) with random primers using a Promega reverse transcription (RT) kit according to the manufacturer’s instructions. No-reverse-transcriptase control reactions, which lacked reverse transcriptase enzyme in the RT reaction mixture, were also performed for all samples. After RT, the reaction mixture was diluted with 30 μl of nuclease-free water and 3 μl of this reaction mixture was used in a 10-μl reaction volume for total quantitative PCRs (qPCRs) according to manufacturer’s instructions using a Microamp fast optical 96-well reaction plate with barcode and an ABI Fast 7500 real-time PCR system (Applied Biosystems) in fast mode with Fast SYBR green master mix (Applied Biosystems). Primers sets used for each gene and *orf10B* internal control, the latter expressed equally under GL and RL conditions (69), are listed in Table SA2 in the supplemental material. The annealing/extension temperature for all primer sets was 60°C, and all primers were verified to produce a single product by melting curve analysis. The abundance of transcripts was determined based on relative quantification levels with normalization to the *orf10B* reference transcript. All qPCR experiments were performed with three independent biological replicates and three technical replicates for each biological replicate. All qPCR procedures and analyses were performed according to the Minimum Information for Publication of Quantitative Real-Time PCR Experiments (MIQE) guidelines (70).

### Measurement of reactive oxygen species.

Reactive oxygen species (ROS) and other peroxide levels were measured using the fluorescent dye 2′,7′-dichlorodihydrofluorescein diacetate (DCF-DA; EMD Chemicals, Gibbstown, NJ) according to previously described methods (44, 71). In brief, aliquots of cells were collected immediately after dilution to a starting OD_750_ of 0.05 (day 0) and after 72 h (day 3) under the desired growth conditions. In a dark room, aliquots were incubated with DCF-DA (10 µM [final concentration]) for 1 h at room temperature with rocking. Fluorescence measurements were then taken at 520 nm with excitation at 485 nm, using water for blanking. The measurements were normalized by the OD_750_ of the culture and are directly proportional to total hydroxyl groups in the sample.

### Protein extraction.

After 7 days of growth under the desired condition, cells were harvested by centrifugation at 5,125 × *g* and 4°C for 10 min. Spent medium was decanted, and then pellets were resuspended in the remaining media (~200 µl) and transferred to 1.5-ml microcentrifuge tubes. Cells were then centrifuged at 16,000 × *g* at 4°C for 5 min, the remaining medium was aspirated, and the cell pellet mass was recorded using a Mettler Toledo XS104 analytical balance (Mettler Toledo, Columbus, OH). The pellets were resuspended in 20 mM Tris-HCl (pH 7.5) with 0.6 M sucrose (33), 0.2 mg/ml (wt/vol) lysozyme, 1× Protease Arrest (G Biosciences, St. Louis, MO), and 5 mM EDTA, at a ratio of 6 ml buffer per gram of cell paste, and were transferred to 15-ml Falcon tubes. Samples were passed through a prechilled French pressure cell press (SLM Instruments, Inc., Urbana, IL) at 500 lb/in^2^ for a total of three times per sample. Each sample was collected in a 15-ml Falcon tube and then transferred into 1.5-ml microcentrifuge tubes and centrifuged at 16,000 × *g* at 4°C for 5 min. Following collection of the soluble fraction, the cell pellet was resuspended to the original volume using 20 mM Tris buffer to obtain a resuspended insoluble fraction at a concentration nearly equal to that of the obtained soluble fraction.

### Quantitative Western analysis.

Prior to SDS-PAGE, total protein concentrations of soluble lysates were measured using the bicinchoninic acid (BCA) assay (Pierce BCA protein assay kit; Thermo Fisher Scientific, Waltham, MA) following the manufacturer’s recommendations. Lysates were then normalized to total protein levels, with addition of 20 mM Tris (pH 7.5) containing 0.6 M sucrose where needed. Samples normalized to total protein levels were then diluted with 5× SDS sample buffer, and then a 2-fold dilution series, up to a 32-fold dilution, was conducted using 1× SDS sample buffer. Insoluble fractions and whole-cell pellets were resuspended in 1× SDS before loading. Samples were denatured at 95°C for either 1 min (soluble fractions) or 5 to 10 min (insoluble fractions).

Proteins (with expected kilodalton values for monomers shown in square brackets) were separated on Tris-HCl gels with 10% acrylamide (CcmM [35 and 60 kDa for the short and long isoforms, respectively] or RbcL [53 kDa]) or 15% acrylamide (CcmK2 [11 kDa]) using Tris-glycine SDS running buffer. After separation by electrophoresis, proteins were transferred to an Immobilon-P polyvinylidene difluoride (PVDF) membrane (EMD Millipore, Billerica, MA) using a semidry transblot Turbo transfer system (Bio-Rad, Hercules, CA) at 25 V (1.0 A max) for 40 min. PVDF membranes were blocked for 1 h at room temperature using 5% (wt/vol) dry milk in Tris-buffered saline (TBS) with 0.5% (vol/vol) Tween 20. After blocking, PVDF membranes were probed using polyclonal rabbit antiserum raised against *Synechococcus* PCC 7942 CcmK2 (72) or CcmM (66) (anti-Ccm antibodies were provided by Cheryl Kerfeld), as well as antiserum raised against *Spinacia oleracea* RbcL (AS07 218, lot 1004; AgriSera, Vännäs, Sweden). Primary antibody incubation was performed up to overnight at 4°C. Blots were washed four times for 10 min each time in TBS–0.1% (vol/vol) Tween (TBS/T) before addition of goat anti-rabbit, horseradish peroxidase (HRP)-conjugated secondary antibody at a dilution of 1:20,000 for 1 h at room temperature. Following four 10-min washes in TBS/T and two 5-min washes in TBS, HRP signal was detected using Femto Glow Western Plus HRP substrate (Michigan Diagnostics, Royal Oak, MI) and a ChemiDoc XP (Bio-Rad, Hercules, CA) imaging system.

### Densitometry analysis.

Densitometry was performed using ImageLab (Bio-Rad, Hercules, CA) software. Lanes were manually selected, and bands were detected using high sensitivity, discarding bands that clearly represented staining artifacts. The disc size, which determines the baseline, was set such that it reliably connected the bases of nonoverlapping peaks (typically, this was a disc size of 10 to 20 mm). Using the same method, total protein was analyzed using Coomassie-stained gels run in parallel to Western blots. The ratio of total protein in the WT strain to that in the Δ*rcaE* strain was analyzed for each dilution factor and found to be nearly 1 in the linear range.

### Purification of *F. diplosiphon* CcmM (*Fd*CcmM) after expression in *Escherichia coli.*

Primers for *ccmM* from *F. diplosiphon* were designed with overhanging restriction sites such that the PCR fragment could be introduced into pET28a to add either an N-terminal 6× His tag (using restriction sites for NheI and XhoI) or a C-terminal 6× His tag (using restriction sites for NcoI and XhoI). After standard cloning methods and bacterial transformation, *E. coli* BL21 strains containing each of the two constructs were analyzed to confirm fragment insertion. Expression of *ccmM* was induced overnight at 30°C using 0.5 mM isopropyl β-D-1-thiogalactopyranoside (IPTG). Cell pellets were harvested, resuspended in 15 ml of native binding buffer (50 mM NaH_2_PO_4_, 0.5 M NaCl, pH 8.0), and passed two times through a CF Range Cell disruptor (Constant Systems Ltd., Daventry, Northamptonshire, United Kingdom) operated at 15,000 lb/in^2^ in a cold (4°C) room. Lysate was then spun at 5,125 × *g* for 15 min at 4°C, and the soluble fraction was extracted and incubated with nickel-nitrilotriacetic acid (Ni-NTA) for 1 h in a purification column (Invitrogen Life Technologies, Inc., Carlsbad, CA). Affinity chromatography was performed according to manufacturer’s instructions, and bound protein was eluted using native binding buffer containing 250 mM imidazole. SDS-PAGE analysis was used to identify elution fractions containing purified CcmM.

### Statistical analysis.

All experiments included at least three independent biological replicates, and results are presented as mean values (± SDs). Statistical analyses were conducted using a Welch two-sample *t* test performed in R (73). The significance level was set at 0.05 for all statistical analyses.

10.1128/mSphere.00617-17.2 FIG S1  Growth curve of *Fremyella diplosiphon* wild-type (WT) and Δ*rcaE* strains under white light. Growth rates of the WT and Δ*rcaE* strains in ambient air were estimated using optical density at 750 (OD_750_) measured once every 24 h for 7 days. Data points represent averages (± standard deviations). *, *P* < 0.05 (for results of comparisons between the Δ*rcaE* and WT strains determined using an unpaired *t* test). Download FIG S1, PDF file, 0.04 MB. Copyright © 2018 Rohnke et al. 2018 Rohnke et al. This content is distributed under the terms of the Creative Commons Attribution 4.0 International license.

10.1128/mSphere.00617-17.3 FIG S2  Highlighted regions represent exclusive peptides of the 30-kDa band identified in anti-CcmM immunoblots which map to the *Fremyella diplosiphon* CcmM sequence. Nine exclusive unique peptides and 12 exclusive unique spectra were identified among 86 total spectra. The identified peptides map 152/552 amino acids (i.e., 28% coverage) of full-length CcmM. Download FIG S2, PDF file, 0.1 MB. Copyright © 2018 Rohnke et al. 2018 Rohnke et al. This content is distributed under the terms of the Creative Commons Attribution 4.0 International license.

10.1128/mSphere.00617-17.4 FIG S3  Quantitative PCR (qPCR)-based gene expression analyses in WT, Δ*rcaF*, and Δ*rcaC* strains of *Fremyella diplosiphon*. Expression levels of *ccmK2* and *ccmM* genes in WT and Δ*rcaF*, and Δ*rcaC* strains grown under green light (GL) or red light (RL) are shown. Expression data for genes are presented relative to the internal control *orf10B*, and the data in each panel are shown relative to expression of the gene of interest in WT cells under GL conditions. Bars represent averages (± SD) of data from three independent biological replicates. Identical letters over bars represent homogenous mean groups (*P* > 0.05); different symbols indicate a statistically significant difference (*P* < 0.05) from others. Download FIG S3, PDF file, 0.04 MB. Copyright © 2018 Rohnke et al. 2018 Rohnke et al. This content is distributed under the terms of the Creative Commons Attribution 4.0 International license.

10.1128/mSphere.00617-17.5 FIG S4  Negative staining via transmission electron microscopy (TEM) of whole cells of a *Synechococcus* sp. PCC 7942 wild-type (WT) strain and a carboxysome-deficient Δ*ccmK2*-*ccmO* strain grown under white light (WL) conditions. To assess whether the electron-dense bodies observed in negative-stained whole cells were carboxysomes or other subcellular structures, we compared TEM images of (a) the WT strain, which was grown in ambient air, and (b) a carboxysome-deficient Δ*ccmK2-ccmO* strain, which has a high-carbon-requiring growth phenotype and thus was grown in 3% CO_2_. Download FIG S4, PDF file, 0.4 MB. Copyright © 2018 Rohnke et al. 2018 Rohnke et al. This content is distributed under the terms of the Creative Commons Attribution 4.0 International license.

10.1128/mSphere.00617-17.6 FIG S5  Energy-dispersive X-ray spectroscopy elemental (EDX) analysis in scanning transmission electron microscopy (STEM) mode of negative-stained whole-cell *Fremyella diplosiphon* ∆*rcaE* mutant strain grown under RL. Multiple elements were analyzed for colocalization with electron-dense bodies. (A and C) Phosphate (P). (B) Electron-dense particles seen in STEM analysis. (D) Magnesium (Mg). (E) Potassium (K). The bar represents 2 µm and is applicable to panel A only. Download FIG S5, PDF file, 0.1 MB. Copyright © 2018 Rohnke et al. 2018 Rohnke et al. This content is distributed under the terms of the Creative Commons Attribution 4.0 International license.

10.1128/mSphere.00617-17.7 TABLE S1  RNA sequencing data for polyphosphate synthesis and degradation genes from *F. diplosiphon* SF33 WT and Δ*rcaE* mutant strains grown under GL or RL conditions. Download TABLE S1, PDF file, 0.1 MB. Copyright © 2018 Rohnke et al. 2018 Rohnke et al. This content is distributed under the terms of the Creative Commons Attribution 4.0 International license.

10.1128/mSphere.00617-17.8 TABLE S2  Quantitative RT-PCR (qRT-PCR) primers used in this study. Download TABLE S2, PDF file, 0.03 MB. Copyright © 2018 Rohnke et al. 2018 Rohnke et al. This content is distributed under the terms of the Creative Commons Attribution 4.0 International license.
