# Supplementary material for: RcaE-Dependent Regulation of Carboxysome Structural Proteins Has a Central Role in Environmental Determination of Carboxysome Morphology and Abundance in Fremyella diplosiphon
Source: mSphere. 2018 Jan 24;3(1):e00617-17. doi: 10.1128/mSphere.00617-17 (PMC5784247; doi:10.1128/mSphere.00617-17)
Supplement: TEXT S1 [file sph001182465s1.pdf]

Text S1

<u>Protein sequencing analysis of ~30 kDa band identified from anti-CcmM immunoblot of *F. diplosiphon* CcmM</u>

After electrophoresis of an elution fraction containing purified *F. diplosiphon* CcmM protein expressed in *E. coli* with an N-terminal His tag, gel bands ranging from 28-35kDa were digested as previously described (Shevchenko *et al.*, 1996), with modifications, at the Proteomics Research Technology Support Facility at Michigan State University. Briefly, gel bands were dehydrated using 100% acetonitrile and incubated with 10 mM dithiothreitol in 100 mM ammonium bicarbonate (pH ~8) at 56°C for 45 min, dehydrated again and incubated in the dark with 50 mM iodoacetamide in 100 mM ammonium bicarbonate for 20 min. Gel bands were then washed with ammonium bicarbonate and dehydrated again. Sequencing grade, modified typsin was prepared to 0.01 µg/µL (w/v) in 50 mM ammonium bicarbonate and ~50 µL of this was added to each gel band so that the gel was completely submerged. Bands were then incubated at 37°C overnight. Peptides were extracted from the gel by water bath sonication in a solution of 60%ACN/1%TCA (v/v) and vacuum dried to ~2 µL. Peptides were then re-suspended in 2% acetonitrile/0.1%TFA to 20 µL. From this, 5 µL were automatically injected by a Thermo EASYnLC 1000 (Thermo Fisher Scientific, Waltham, MA) onto a Thermo Acclaim PepMap RSLC 0.075 mm x 250 mm C18 column and eluted over 16 min with a gradient of 5% Buffer B (i.e., B; Buffer B = 99.9% Acetonitrile/0.1% Formic Acid) to 30% B in 1 min, ramping to 90% B at 2 min and held at 90% B for the duration of the run at a constant flow rate of 300 nl/min. Buffer A = 99.9% Water/0.1% Formic Acid.

Eluted peptides were sprayed into a ThermoFisher Q-Exactive mass spectrometer (Thermo Fisher Scientific, Waltham, MA) using a FlexSpray spray ion source. Survey scans

were taken in the Orbi trap (35000 resolution, determined at m/z 200) and the top ten ions in each survey scan are then subjected to automatic higher energy collision induced dissociation (HCD) with fragment spectra acquired at 17,500 resolution. The resulting MS/MS spectra are converted to peak lists using Mascot Distiller, v2.6 (Matrix Scientific, Boston, MA) and searched against a database containing all cyanobacteria protein sequences and all E.coli protein sequences available from NCBInr (downloaded 2017-07-07 from www.ncbi.nlm.nih.gov) appended with common laboratory contaminants (downloaded from www.thegpm.org, cRAP project) using the Mascot searching algorithm, v 2.5. The Mascot output was then analyzed using Scaffold, v4.8.2 (www.proteomesoftware.com) to probabilistically validate protein identifications. Assignments validated using the Scaffold 1%FDR confidence filter are considered true.

Electron Dispersive X-ray (EDX) Transmission Electron Microscopy (TEM) analysis

EDX analysis was performed both on conventional TEM sections and negative-stained samples using a JEM-2200FS TEM (JEOL USA Inc., Peabody, MA) with an in-column energy filter operated in Scanning Transmission Electron Microscopy (STEM) mode at 200 kV. The analytical work was done with the attached Oxford Instrument INCA system with energy resolution of 140 eV. The images were collected with a Gatan Multiscan camera at 1024x1024 resolution.

Reference

Shevchenko, A., Wilm, M., Vorm, O. and Mann, M. (1996) Mass spectrometric sequencing of proteins from silver-stained polyacrylamide gels. *Anal Chem* **68**: 850-858.
