# Supplementary figures and images for: RcaE-Dependent Regulation of Carboxysome Structural Proteins Has a Central Role in Environmental Determination of Carboxysome Morphology and Abundance in Fremyella diplosiphon
Source: mSphere. 2018 Jan 24;3(1):e00617-17. doi: 10.1128/mSphere.00617-17 (PMC5784247; doi:10.1128/mSphere.00617-17)

Figure S1

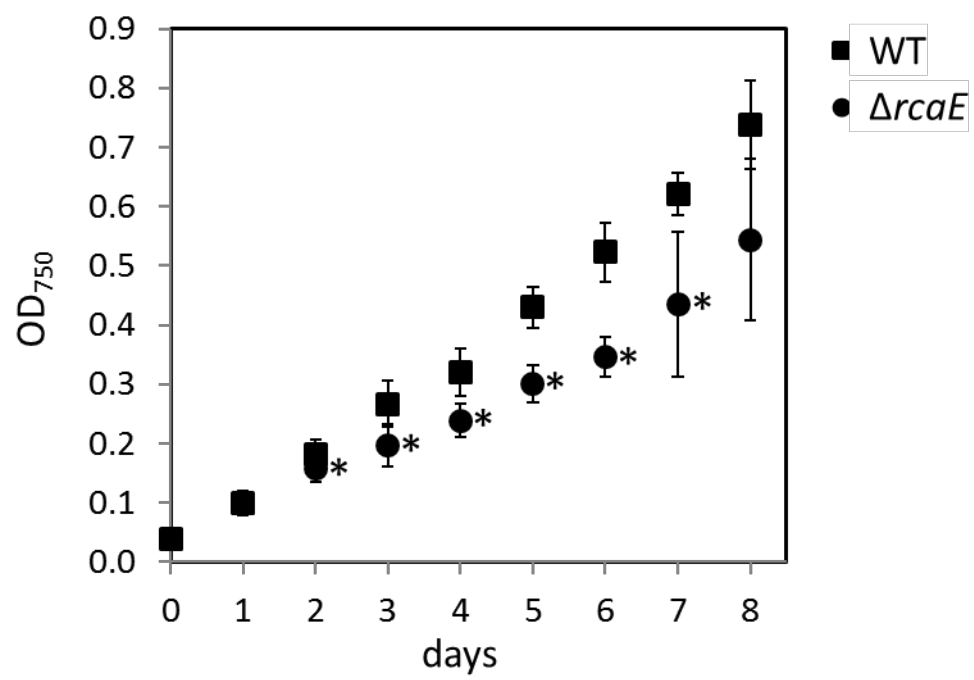

Supplement: FIG S1 [file sph001182465sf1.pdf]

**Figure S3**

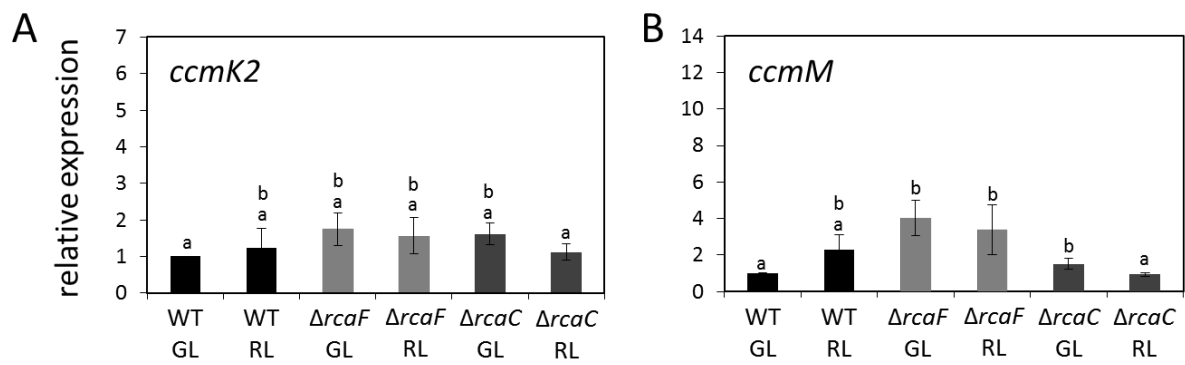

Supplement: FIG S3 [file sph001182465sf3.pdf]

**Figure S4**

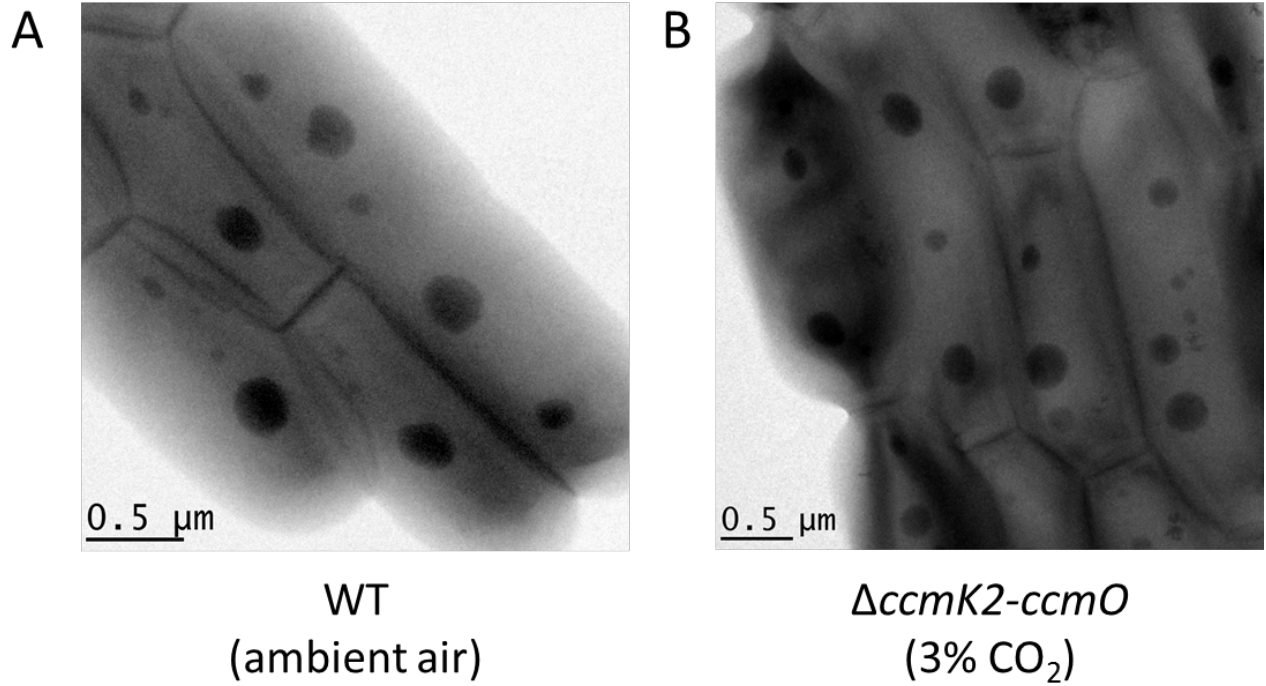

Supplement: FIG S4 [file sph001182465sf4.pdf]

**Figure S5**

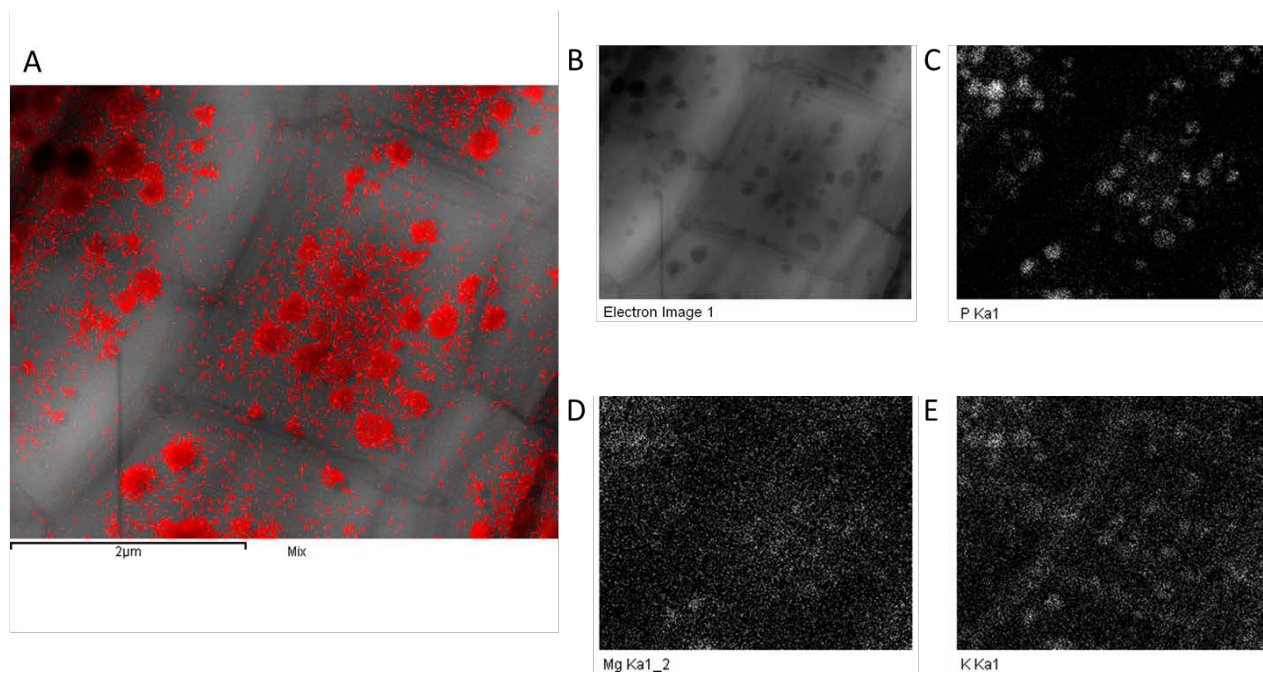

Supplement: FIG S5 [file sph001182465sf5.pdf]
