# Supplementary material for: RcaE-Dependent Regulation of Carboxysome Structural Proteins Has a Central Role in Environmental Determination of Carboxysome Morphology and Abundance in Fremyella diplosiphon
Source: mSphere. 2018 Jan 24;3(1):e00617-17. doi: 10.1128/mSphere.00617-17 (PMC5784247; doi:10.1128/mSphere.00617-17)
Supplement: FIG S2 [file sph001182465sf2.pdf]

Figure S2

M**AVSSTVAPP** **TPWSRNLAEP** **TIHESTFVHS** **FSK**VIGDVR**I** **GANVIVAPGT**
SIRADEGTPF FIGENTNIQD GVVIHGLEQG RVIGDDQEEY SVWIGKNASI
THMALIHGPA YVGDNSFIGF RSTVFNARVG AGCIVMMHAL IQDVEIPPGK
YVPSGAIITT **QQQADRLPDV** **QSQDQEFTHH** **VVGINQALR**A GYLCVADSKC
ITPIRDQIKK SSTGNGITVL ELER**SSEVAS** **GSLSAETIEQ** **VRYLLQQGYK**
IGTEHVDQRR FRTGSWTSCQ PINARSIGEA ISALEACLAD HAGEYVRLFG
IDNGRRRVLE TIIQRPDGTV GAPAAAFKAP KAASNGSYSS NGNGNGSGGG
LNTETVEQIR QLLAGGYK**IG** **TEHVDERR**FR TGSWQSCSPI NSTSTNEVIA
ALEDCISNHQ GEYVRLIGID PKAKRRVLES IIQRPNGQVN PSSSTK**SFTS**
SAPTATATAT **ATATSTRLSG** **EVIDQLR**QLL GAGYKISVEH VDQRRFRTGS
WTSTGPIQAN SERDAIAAIE AALAEYAGEY VRLIGIDPKA KRRVLETIIQ
RP
