# Supplementary material for: RcaE-Dependent Regulation of Carboxysome Structural Proteins Has a Central Role in Environmental Determination of Carboxysome Morphology and Abundance in Fremyella diplosiphon
Source: mSphere. 2018 Jan 24;3(1):e00617-17. doi: 10.1128/mSphere.00617-17 (PMC5784247; doi:10.1128/mSphere.00617-17)
Supplement: TABLE S1 [file sph001182465st1.pdf]

Table S1. RNA sequencing data for polyphosphate synthesis and degradation genes from *F. diplosiphon* SF33 WT and Δ*rcaE* mutant strains grown under GL or RL conditions.

Gene	*Ava*[a] homolog	No. Reads				Fold change[b] RL vs. GL		Fold change[c] Δ*rcaE* vs. WT	
		WT		Δ*rcaE*		WT	Δ*rcaE*	GL	RL
		GL	RL	GL	RL				
ppk	Ava_3165	473.5	349	558.5	536	0.7	0.96	1.2	1.53
ppx	Ava_3530	410	298	408	427	0.72	1.04	0.995	1.4

[a] ORFs were compared against *Anabaena variabilis* (Ava) ATCC 29413 annotated proteins using BlastX with a cut-off e-value of 0.0001 to determine Ava homolog.

[b] Fold change, differential expression analysis between two light treatments was calculated for each strain. Note: no significant differences were detected using *t*-test.

[c] Fold change, differential expression analysis between two strains was calculated for each light condition. Note: no significant differences were detected using *t*-test.
