# Supplementary material for: RcaE-Dependent Regulation of Carboxysome Structural Proteins Has a Central Role in Environmental Determination of Carboxysome Morphology and Abundance in Fremyella diplosiphon
Source: mSphere. 2018 Jan 24;3(1):e00617-17. doi: 10.1128/mSphere.00617-17 (PMC5784247; doi:10.1128/mSphere.00617-17)
Supplement: TABLE S2 [file sph001182465st2.pdf]

Table S2. Quantitative RT-PCR (qRT-PCR) primers used in this study

**Primer name**	**Forward primer/FP (5’-3’)**	**Reverse primer/RP (5’-3’)**
ccmK1	AACGAATTGGCAGGACATACT	GCAGGCGTAGAATCTGTGAA
ccmK2	AGGCTTGCACTTCCGATAC	TGCTGATGCGATGGTGAA
ccmL	GTCTACTCCTGCACCTACGATA	GTCTTCGAGGTGTGAAACTACTG
ccmM	GATTGCTCCCGAAGGTACATATT	GGCTTTCGCTCTACGGTATTT
ccmN	TGGCACTCAGATTTATGGTACAG	GTCCGAGATGGGTTCATTTAGAG
ccmO	CCATTACCTCCAAGCTCAGTAAA	CTCCTACCATCGCTGGAAATC
ccmK3	TGCTGCTGGAGAACAAGTAAA	GTAAAGTGGATCGGAAGGATGG
ccmK4	CAGGCAGTTGGAGCATTAGA	TCAGAAACATCGCCACGAATA
orf10B	AGAACTACAGCGTCAGCTTAAT	CTGCTTCGCTTTCAGCATTT
